# A Rare Presentation of Lichen Sclerosus Involving the Vaginal Canal After Radiation Therapy: A Case Report and Review of the Literature

**DOI:** 10.7759/cureus.47077

**Published:** 2023-10-15

**Authors:** Arcole Brandon, Tanvi Joshi, Zan Ahmed, Muhammad Tahir, Thuy Phung, Jennifer Y Pierce, Kurt Knowles

**Affiliations:** 1 Medicine, University of South Alabama College of Medicine, Mobile, USA; 2 Obstetrics and Gynaecology, University of South Alabama College of Medicine, Mobile, USA; 3 Pathology and Laboratory Medicine, University of South Alabama Health University Hospital, Mobile, USA

**Keywords:** lichen sclerosus clinical manifestasions, vaginal lichen sclerosis, post radiation lichen sclerosus, female lichen sclerosis, lichen sclerosus

## Abstract

Vaginal lichen sclerosus (LS) is an extremely rare entity. Classically, LS is referred to as a chronic, inflammatory skin disease with a distinct predilection for the anogenital skin that is observed in post-menopausal women and typically manifests clinically as white, atrophic plaques. Here, we report a case of a 61-year-old patient who presented for a follow-up visit three years after vaginal brachytherapy as an adjuvant treatment for endometrial adenocarcinoma. This lesion was biopsied and confirmed to be vaginal LS on histological analysis. While LS has been previously observed to impact mucosal areas outside of the anogenital region, such as the mouth, reported cases of vaginal LS are very rare in the literature. Our case highlights both the underrecognized location of this disease as well as radiation as a potential risk factor.

## Introduction

Lichen sclerosus (LS) is a chronic, inflammatory disorder that can lead to scarring, loss of normal architecture of external genitalia, sexual dysfunction, and even malignancy [1]. While LS can be found in both men and women, it primarily tends to affect the female anogenital epithelium [1]. The etiology of LS remains unclear, but some reports suggest that it may involve an autoimmune and genetic component [2]. Physical examination generally reveals white, atrophic-appearing plaques with epidermal thinning (also termed: “cigarette paper” appearance), hyper- or hypo-pigmentation, resorption of the labia minora, clitoral phimosis, and narrowing of the introitus [1]. LS is described as primarily an epithelial disease and is thought to rarely involve the vaginal mucosa [1]. There are only a few reported cases of vaginal or urethral LS [3,4], indicating that vaginal LS is either truly a rare entity or is grossly underreported and/or underdiagnosed. Moreover, in patients with prior radiation exposure, reports of vaginal LS are even more lacking, and, therefore, this case may help shed further insight into radiation as a possible risk factor for LS.

## Case presentation

A 61-year-old patient, with a history of chronic obstructive pulmonary disease (COPD), hypertension, obesity, and hypercholesterolemia, presented three years after her treatment for endometrial cancer with post-coital vaginal bleeding and dyspareunia. Of note, the patient had initially undergone an exam under anesthesia, robotic-assisted total laparoscopic hysterectomy (RA-TLH), and bilateral salpingo-oophorectomy (BSO) for persistent vaginal pain. Pathology was incidentally positive for grade 1 endometrioid adenocarcinoma with stromal invasion of the cervix and >50% myometrial invasion. She was subsequently referred to gynecologic oncology and opted for complete surgical staging via a laparoscopic bilateral sentinel lymph node biopsy. Her final diagnosis was a stage II, grade 1 endometroid adenocarcinoma for which she completed adjuvant vaginal brachytherapy and declined whole pelvic radiation.

At her three-year surveillance visit, the patient complained of post-coital bleeding. A pelvic exam at this time revealed normal external female genitalia with papillary, friable tissue adjacent to the vaginal cuff. This mass was biopsied with Tischler forceps and was noted to be hemostatic with the use of silver nitrate.

**Figure 1:**
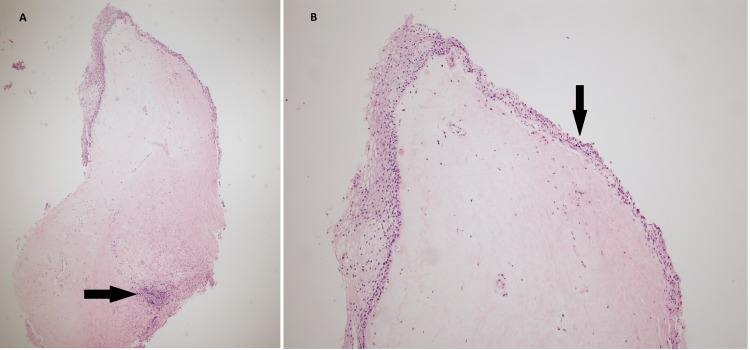
(A) Low power view (4x), demonstrating lichen sclerosus of the vagina evidenced by the widespread hypocellular edematous zone bounded by thin, atrophic epithelium on the surface and inflamed stroma showing chronic inflammatory infiltrate (arrow). (B) Medium power view (10x) highlighting a thin atrophic surface epithelium (arrow); the opposite side reveals normal stratified squamous epithelium.

**Figure 2:**
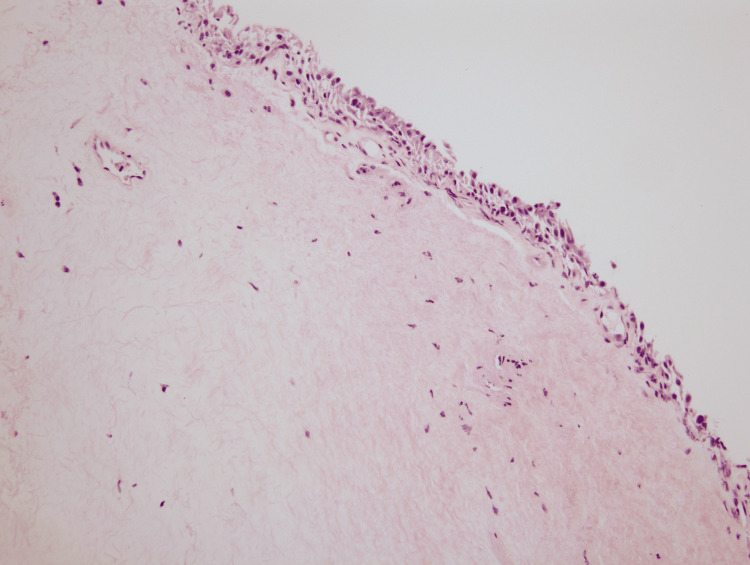
High-power review (20x) of the vaginal biopsy demonstrating eosinophilic homogenization of the collagen in the subepithelial layer, including edema and chronic inflammatory infiltrates. A minor component of the vacuolar interface reaction pattern can be appreciated below the basal epithelium.

The biopsy of the lesion was sent for pathology. Under the microscope at low power view, the overall morphology demonstrated chronic irritational changes with homogenization of the subepithelial compartment with deep sclerosis. A minor focus of perivascular lymphoplasmacytic infiltrate was noted in addition to a bounded layer of atrophic surface epithelium (Figures 1-2). The surface epithelium lacked any atypia of significance. In conjunction with histopathology, a final diagnosis of LS was rendered. The findings in this location are highly unusual and are typical of a vulvar process. However, in view of prior radiation history to this site, radiation-induced LS was considered an alternative diagnosis. Lichen planus, lichen simplex chronicus, and squamous cell carcinoma were also considered but ruled out given tissue morphology. The slide was sent for outside consultation by an expert dermatopathologist who agreed with the above interpretation.

Considering this diagnosis, the patient was started on vaginal suppositories of cortisone initially. She was unable to use these successfully due to persistent dyspareunia within three months of her initial diagnosis of vaginal LS. Therefore, the patient was then started on clobetasol per vagina with the use of a syringe applicator and vaginal estrogen cream. At the most recent visit, the patient continues to tolerate the above therapy and appears to have improved sexual function. On physical examination, the papillary, friable tissue notable on exam remains stable and will continue to be closely monitored with pelvic examinations every three months.

## Discussion

LS, also called lichen sclerosus et atrophicus or chronic atrophic vulvitis, is a chronic inflammatory skin disease that affects the anogenital area in 85-95% of cases [1,5]. While there are pathognomonic features on physical exam, a biopsy can confirm this disorder histologically. On clinical presentation, LS can affect a singular region or the entire region of the vulva, anogenital skin, and even extend to the genitocrural folds and buttock region [5,6]. The characteristic findings include well-demarcated erythema, tissue fragility with erosions, fissuring and purpura, loss of normal labial architecture, clitoral phimosis, and a “crinkling appearance” of the genital skin [7]. LS generally has a bimodal age distribution and can affect both premenopausal girls and menopausal women [1,8].

Histologically, the diagnosis of LS includes a demonstration of a vacuolar interface reaction pattern at the basal epidermal layer in conjunction with dermal sclerosis. The subepithelial compartment becomes homogenized with a further appreciation of hyalinized eosinophilic collagen bundles admixed with chronic inflammation [9]. The overlying epidermis is characteristically atrophic, but, on occasion, it may show foci of hyperplasia [10].

Vaginal involvement by LS is an uncommon entity with less than seven reported cases in the literature, as demonstrated below in Table 1. The etiology is unclear, but it is thought that autoimmune and genetic factors may predispose to its development. In other circumstances, chronic inflammation may lead to squamatization of the epithelium, which can later progress to LS [5]. While LS has now been accepted as a risk factor for the development of vulvar precancerous lesions and squamous cell carcinoma, the risk of neoplasia with vaginal LS is not well defined [1]. Therefore, it is imperative to closely surveil patients with this diagnosis and be liberal with the decision to obtain another biopsy as indicated for clinical changes.

**Table 1:** Summarizing the six cases of lichen sclerosus, the risk factors, histopathological findings, and treatment.

Reported Cases in the Literature of Vaginal Lichen Sclerosus				
Patient Age/Race	Risk Factors	Lesion Location	Histopathological Characteristics	Treatment
61/Unknown Race, Xavier et al. [3]	She had a prior surgical correction of a cystocele. Grade I cystocele was noted at the time of the vaginal LS diagnosis.	The outer third of the vagina, including the anterior vaginal wall	Hyperkeratosis, subepithelial hyalinization of collagen, and deep lymphocytic inflammatory infiltrates were all noted in the biopsy.	Topical clobetasol 0.05%, emollients, hydroxyzine, and twice a week topical vaginal estrogen were used with significant symptom improvement noted after two months.
60/Unknown Race, Xavier et al. [3]	When she presented, she had a two-year history of biopsy-confirmed lichen sclerosus of the vagina that had not yet responded to topical estriol, clobetasol, and emollients. She had a hysterectomy and bilateral salpingo-oophorectomy at age 52 for a benign disease.	Posterior wall of the vagina	She was only noted to have a prior vaginal biopsy with a histological result of lichen sclerosus.	She was initially started on pimecrolimus cream 0.3% twice daily with no improvement noted. Clobetasol propionate and subcutaneous administration of triamcinolone provided some improvement of the symptoms. Eventually, the patient was started on retinoids with improvement of the symptoms and vulvovaginal lesions.
54/White, Longinotti et al. [4]	She had a four-year history of vulvar lichen sclerosus unresponsive to triamcinolone intravaginal cream, vaginal estradiol tab therapy (discontinued due to chronic vaginal wetness), clobetasol propionate ointment 0.05% topically, and 1% hydrocortisone cream, but she did report some relief from 3% testosterone in Aquaphor. She had a hysterectomy at the age of 23 for a benign disease.	Anterior wall of the vagina below the urethra at the level of the vaginal vault (about seven to eight cm above the introitus) extending to the posterior vaginal fornix	It was notable for thinning of the squamous epithelial lining with pink staining acellular upper stroma, beneath which lay an inflammatory infiltrate of lymphocytes and plasma cells, and vascularization can be noted within the basal layer of the cells. The dermis demonstrates an acellular, homogenous appearance. There is an absence of elastic fibers.	No treatment was used for the vaginal LS. For the vulvar LS, topical clobetasol ointment 0.05% applied to the vulva twice daily for one month, at bedtime for two months, and every other day for three months was used, and at a follow up visit, the vulvar and vaginal lichen sclerosus were noted to be unchanged, but the patient was now asymptomatic.
76 (at the initially described presentation)/White, Zendell et al. [5]	She had a history of a rectocele. She also had a five-year prior history of what became clinically diagnosed as vulvar lichen sclerosus with involvement of the labia minora and the perineal body previously unresponsive to vaginal estrogen cream, topical and vaginal antifungal creams, topical corticosteroid creams, and hydroxyzine hydrochloride.	Distal posterior vaginal wall	Compact hyperkeratosis, epithelial atrophy, focal vacuolization along the basement membrane zone, and hyalinization of the mucosal subepithelium with a thickened basement membrane and extravasated erythrocytes present focally below the zone of hyalinization were all noted.	Treatment of her vaginal lichen sclerosus was not described in this case report. Vulvar LS was treated with ultrapotent topical corticosteroid ointments and vaginal estrogen cream with “waxing and waning” success.
59 (at the initially described presentation)/White, Zendell et al. [5]	She had a history of a rectocele and “marked pelvic organ laxity”. She also had a history of several years of difficulty controlling LS located on her upper back, inner thighs, vulva, perineum, and perineal body. She also had multiple noted but unspecified socioeconomic risk factors. Post-menopausal atrophy of the vagina was noted on the initial exam before diagnosing LS of the vagina.	Distal anterior vaginal wall	Hyperkeratosis overlying the mucosal epithelium with hyalinization of the subepithelium.	Topical corticosteroids three times weekly and vaginal estrogen therapy were used with “fairly good control” of the disease noted.
59/Unknown Race, Bhargava et al. [6]	She had a history of grade II uterine prolapse and a cystocele leading to pain and urinary incontinence.	The location of the vaginal LS is referred to in the region of “the exposed prolapsed vaginal wall secondary to the cystocele, visible at the introitus”, but no specific location is mentioned	A biopsy does not appear to have been performed. Clinical changes were described as “keratinized” with “patchy and confluent smooth porcelain white patches with conspicuous purpura” on the exposed, prolapsed vaginal wall.	Clobetasol propionate 0.05% ointment for three months with emulsifying ointment as a soap substitute was used which resulted in resolution of her pruritis. The anterior repair of her uterine prolapse and cystocele led to a marked improvement in her urinary symptoms.

Interestingly, our case also highlights prior radiation as a potential risk factor for the development of vaginal LS. Radiation-induced vulvar LS was identified in one case report in a woman with prior external pelvic radiation and brachytherapy for vaginal cancer [11]. However, most cases of radiation-induced LS have been reported as extragenital LS, such as with radiation to the breast [12,13]. Importantly, this highlights that LS can preferentially arise in a previously irradiated region. In most reported cases, the time from radiation to the onset of symptomatic LS ranged from two to 12 years [12-14], which corresponds to our patient’s clinical presentation. This shows that radiation may indeed lead to changes in the epithelium and mucosal surfaces and, therefore, potentially induce LS development.

## Conclusions

In conclusion, vaginal LS is an unusual presentation with less than seven reported cases in the literature. Our case underscores the importance of not only recognizing this diagnosis in the vaginal canal but also highlights the potential role of radiation as a risk factor. While the risk of subsequent progression to cancer is not well-established, precaution should be maintained to follow up patients with this diagnosis clinically, and, if any examination changes are noted, one should be especially vigilant about obtaining a histopathological diagnosis.

## References

[1] Krapf Jm, Mitchell L, Holton Ma, Goldstein AT (2020). Vulvar lichen sclerosus: current perspectives. Int J Womens Health.

[2] Sherman V, McPherson T, Baldo M, Salim A, Gao XH, Wojnarowska F (2010). The high rate of familial lichen sclerosus suggests a genetic contribution: an observational cohort study. J Eur Acad Dermatol Venereol.

[3] Xavier J, Vieira-Baptista P, Moreira A, Portugal R, Beires J, Tanos V (2018). Vaginal lichen sclerosus: report of two cases. Facts Views Vis Obgyn.

[4] Longinotti M, Schieffer YM, Kaufman RH (2005). Lichen sclerosus involving the vagina. Obstet Gynecol.

[5] Zendell K, Edwards L (2013). Lichen sclerosus with vaginal involvement: report of 2 cases and review of the literature. JAMA Dermatol.

[6] Bhargava K, Lewis FM (2013). Lichen sclerosus occurring on vaginal mucosa secondary to uterine prolapse. J Obstet Gynaecol.

[7] Kumar V, Abbas AK, Aster JC (2020). Robbins & Cotran Pathologic Basis of Disease. 10th Edition. Elsevier inc.

[8] Fistarol SK, Itin PH (2013). Diagnosis and treatment of lichen sclerosus: an update. Am J Clin Dermatol.

[9] Goldblum JR, Lamps LW, Mckenney JK (2018). Rosai and Ackerman's Surgical Pathology. 11th Edition. Elsevier inc.

[10] Lee Es, Allen D, Scurry J (2003). Pseudoepitheliomatous hyperplasia in lichen sclerosus of the vulva. Int J Gynecol Pathol.

[11] Edwards LR, Privette ED, Patterson JW (2017). Radiation-induced lichen sclerosus of the vulva: first report in the medical literature. Wien Med Wochenschr.

[12] Vujovic O (2010). Lichen sclerosus in a radiated breast. CMAJ.

[13] Petersen E, Yazdani L, Hymes SR (2018). A case of radiation-induced bullous morphea/lichen sclerosus overlap in a breast cancer patient. Rep Pract Oncol Radiother.

[14] Yates VM, King CM, Dave VK Lichen sclerosus et atrophicus following radiation therapy. Arch Dermatol.

